# Circadian and wake-dependent changes in human plasma polar metabolites during prolonged wakefulness: A preliminary analysis

**DOI:** 10.1038/s41598-019-40353-8

**Published:** 2019-03-14

**Authors:** Leilah K. Grant, Suzanne Ftouni, Brunda Nijagal, David P. De Souza, Dedreia Tull, Malcolm J. McConville, Shantha M. W. Rajaratnam, Steven W. Lockley, Clare Anderson

**Affiliations:** 1 School of Psychological Sciences and Monash Institute of Cognitive and Clinical Neurosciences, Monash University, Melbourne, Australia; 2 Cooperative Research Centre for Alertness, Safety and Productivity, Melbourne, Australia; 3 Division of Sleep and Circadian Disorders, Departments of Medicine and Neurology, Brigham and Women’s Hospital, Boston, USA; 4 Division of Sleep Medicine, Harvard Medical School, Boston, USA; 5 Metabolomics Australia, Bio21 Molecular Science and Biotechnology Institute, Parkville, Australia

## Abstract

Establishing circadian and wake-dependent changes in the human metabolome are critical for understanding and treating human diseases due to circadian misalignment or extended wake. Here, we assessed endogenous circadian rhythms and wake-dependent changes in plasma metabolites in 13 participants (4 females) studied during 40-hours of wakefulness. Four-hourly plasma samples were analyzed by hydrophilic interaction liquid chromatography (HILIC)-LC-MS for 1,740 metabolite signals. Group-averaged (relative to DLMO) and individual participant metabolite profiles were fitted with a combined cosinor and linear regression model. In group-level analyses, 22% of metabolites were rhythmic and 8% were linear, whereas in individual-level analyses, 14% of profiles were rhythmic and 4% were linear. We observed metabolites that were significant at the group-level but not significant in a single individual, and metabolites that were significant in approximately half of individuals but not group-significant. Of the group-rhythmic and group-linear metabolites, only 7% and 12% were also significantly rhythmic or linear, respectively, in ≥50% of participants. Owing to large inter-individual variation in rhythm timing and the magnitude and direction of linear change, acrophase and slope estimates also differed between group- and individual-level analyses. These preliminary findings have important implications for biomarker development and understanding of sleep and circadian regulation of metabolism.

## Introduction

Circadian rhythms, endogenously generated cycles of approximately 24 hours, govern many patterns of behavior and physiology including sleep/wake cycles, cognition, feeding patterns, hormone secretion, gene expression and cellular processes. Given the circadian system’s control over so many biological processes, it is unsurprising that disruption to this endogenous clock and its outputs is associated with adverse health outcomes. Shift workers, for example, whose circadian rhythms are often chronically misaligned from their sleep-wake cycle^1,2^, have an increased risk of developing serious diseases including obesity, diabetes, cardiovascular disease, stroke and some cancers^3–5^. Moreover, experimentally-induced circadian disruption in controlled laboratory settings shows that misalignment of circadian and behavioral cycles leads to acute cardiometabolic dysfunction in humans^6–8^. A direct influence of the circadian system on metabolic homeostasis has been demonstrated in rodents, whereby knocking out core clock genes significantly alters metabolism^9–11^. Furthermore, studies have demonstrated 24-h rhythms in the hepatic, serum and plasma metabolomes of rodents^12–14^, prompting investigation of circadian control of the metabolome in humans.

Metabolomic analysis of human plasma samples collected during a normal day with either an 8:16 h sleep/wake cycle, or during sleep deprivation reveals 24-h oscillations in addition to wake-dependent increases or decreases in metabolites from a wide variety of chemical classes^15,16^. Studies conducted under constant routine conditions, the gold-standard method for assessing endogenous circadian rhythms^17^, have also described 24-h rhythms and increases or decreases over time awake in the human metabolome^18–21^. Analysis of individual metabolomic profiles, however, has shown substantial inter-individual differences in the timing and abundance of rhythmic lipids and in the magnitude and direction of change in lipids that increase or decrease with time awake^18,19^. Despite this variability, many of the studies published to date have only conducted group-level analyses, which given the underlying inter-individual variation, may not accurately describe circadian and wake-dependent control of metabolite levels. Furthermore, previous studies have focused on metabolites that are resolved on reverse phase LC matrixes (i.e. capturing lipids, fatty acids, acyl carnitines, some amino acids and carbohydrates) and have not detected changes in more polar compounds, such as nucleotides, nucleosides, organic acids, amino acids, and carbohydrates, which are important intermediates in central carbon metabolism and are reflective of changes in macromolecule synthesis, the urea cycle, and pathways of energy (i.e. glycolysis and the Krebs cycle).

Polar metabolites have been identified as biomarkers of cancers^22,23^, diabetes^24^, Alzheimer’s disease^25^, myocardial ischemia and infarction^26,27^, and osteoarthritis^28^. With single-point assessments of polar metabolites potentially being used as biomarkers of a variety of disease states, it is important that the circadian variation and effect of inadequate sleep on these compounds is well understood. Variation in a metabolite’s concentration at different times of day or variation induced by sleep loss has implications for the timing and interpretation of clinical diagnostic tests and the efficacy of treatments. Improved understanding of circadian- and wake-dependent control of metabolism will also contribute to understanding the etiology of cardiometabolic diseases and may inform future development of interventions and chronotherapies to treat such disorders. In the current study, we characterized circadian- and wake-dependent changes in polar metabolites using HILIC-LC-MS over 40-hours of continuous wakefulness under highly controlled conditions. Changes to metabolite levels were subsequently assessed using both group- and individual-level analyses to observe the degree of concordance between these analysis approaches.

## Results

**Figure 1:**
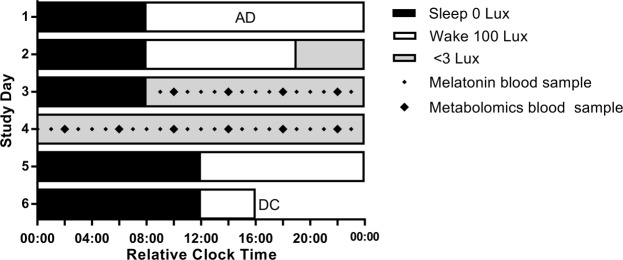
Participants completed a 6-day laboratory protocol. The protocol consisted of (i) two baseline days (8:16 sleep/wake based on average sleep time two weeks before admit [AD]), (ii) a 40-hour constant routine, and (iii) two recovery days with up to 12-hour sleep opportunities before discharge (DC). White bars represent wake episodes in 100 lux, black bars represent sleep episodes in 0 lux, and grey bars represent a DLMO assessment on day 2 and the 40-h CR in <3 lux ambient light. During the CR protocol, black diamonds represent blood samples, with larger diamonds representing samples used in the current metabolomics analysis. The protocol is shown in relative clock time with a relative bedtime of midnight. Study events were scheduled relative to each individual’s pre-study self-selected wake time.

Circadian and wake-dependent modulation of plasma polar metabolites was investigated in 13 healthy adults (4 females) aged 20–32 years (Table 1), who underwent a 40-hour constant routine (CR) protocol (Fig. 1). The final dataset of metabolites included 99 metabolites identified based on their accurate mass and coelution with authentic metabolite standards, in addition to 1,641 unidentified metabolite features that constituted the untargeted matrix and were detected in all participants. Ten of 13 participants had missing data points in the targeted matrix resulting in 14% (18 samples; total n = 112) missing data, and 12 of 13 participants had missing data points in the untargeted matrix resulting in a total of 16% (21 samples; total n = 109) missing data. Further information on missing samples can be found in the methods section.

**Table 1:** Demographic characteristics of study participants.

Demographics	M ± SD or No. (%)
N	13
Age (years)	25.00 ± 4.31
Males	9 (69%)
Body mass index (kg/m^2^)	22.00 ± 2.14
Dim light melatonin onset time (decimal time)	20.91 ± 1.47
Wake time (decimal time)	07.19 ± 0.73
Bed time (decimal time)	23.19 ± 0.73
Morningness Eveningness Questionnaire score	37.92 ± 2.66

*Note:* Participant excluded from the analysis is not included in this table.

**Figure 2:**
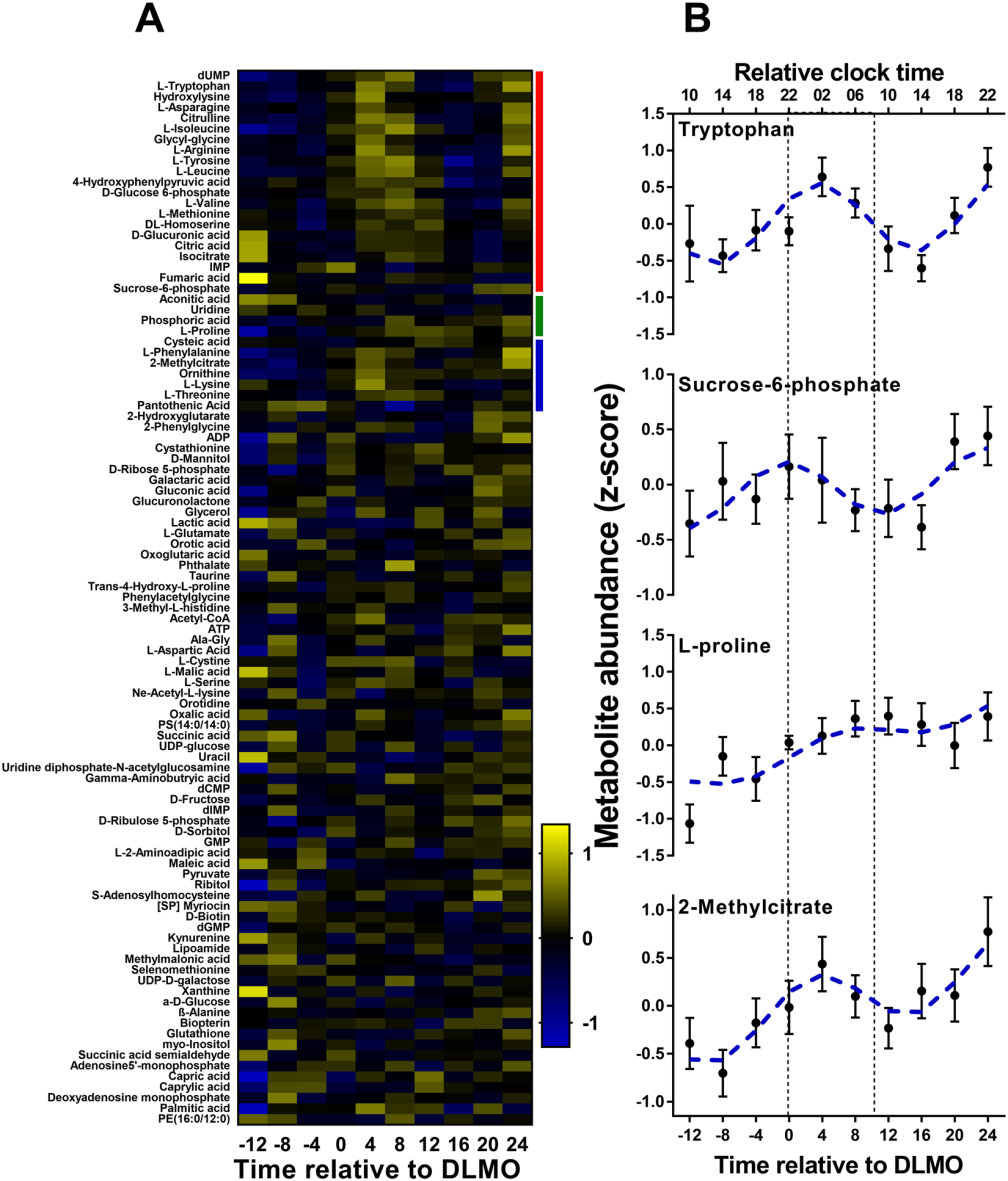
(**A**) Time course of metabolite concentrations (z-score area under the peak) for all identified metabolites in the targeted matrix. Significant metabolites are marked by the coloured bar to the right of the heatmap (red – rhythmic; green – linear; blue – combined rhythmic and linear). Data are represented relative to DLMO (time 0), the time at which plasma melatonin reached 5 pg/mL. (**B**) Examples of significant profiles are shown for tryptophan (*top*: night peaking rhythmic, not linear), sucrose-6-phosphate (*middle, upper:* day peaking rhythmic, not linear), L-proline (*middle, lower:* linear increasing, not rhythmic), and 2-methylcitrate (*bottom:* night peaking rhythmic with linear increase). Data are plotted relative to DLMO, and by relative clock time, with relative bedtime at midnight. Errors bars represent SEM. The area between the dashed lines represent the ‘biological night’, defined as DLMO plus 10 hours, and the blue dashed line represents the predicted fit of the model.

**Figure 3:**
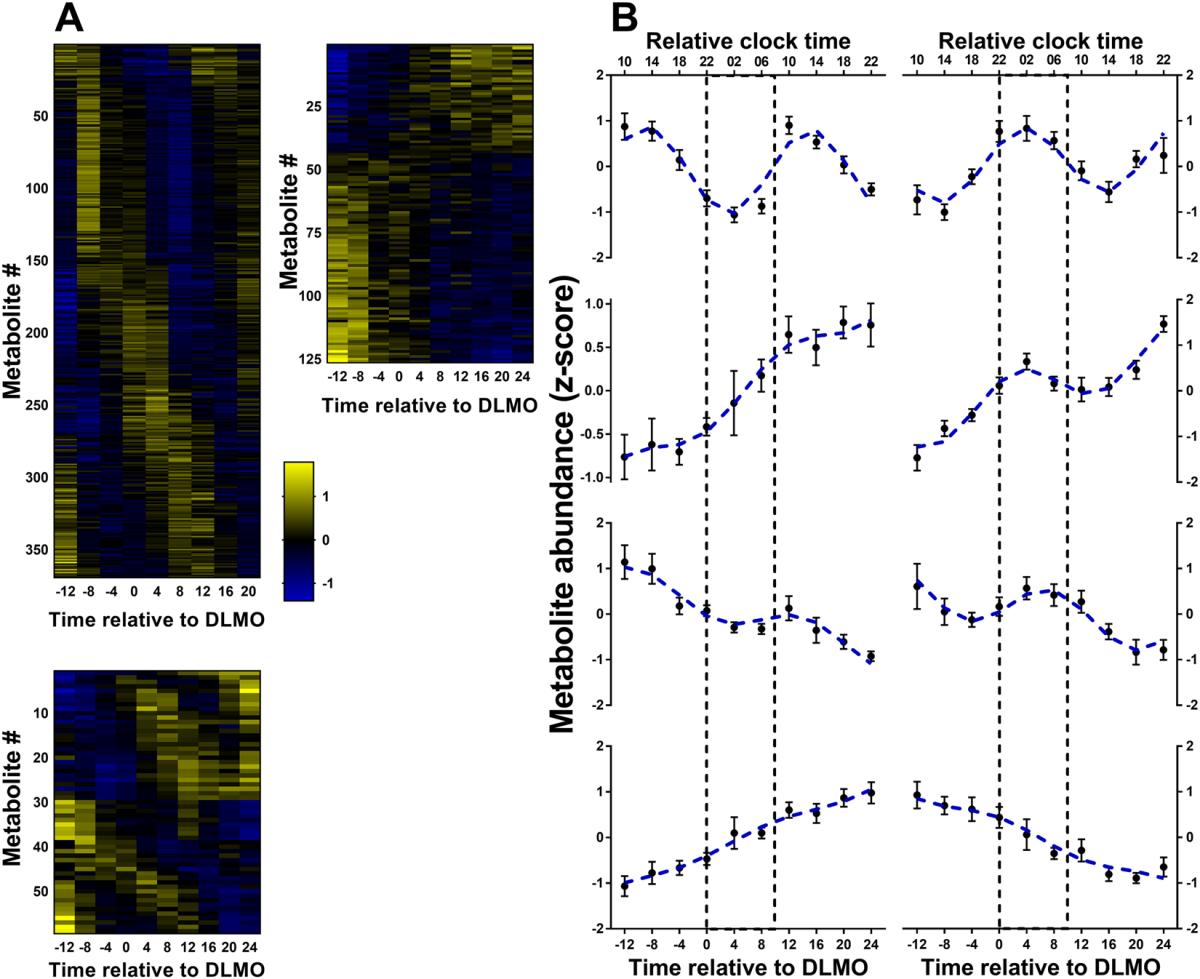
Group-level analysis of the untargeted metabolite matrix. (**A**) The time course of metabolites significantly rhythmic (*top left*), linear (*top right*), and combined rhythmic and linear (*bottom*) in group-level analysis. Data are represented relative to DLMO (time 0), the time plasma melatonin reached 5 pg/mL. (**B**) Metabolite concentrations showing rhythmic and linear trends during sleep deprivation from the untargeted matrix. Examples include day peaking rhythmic, not linear (*top left*), night peaking rhythmic, not linear (*top right*), day peaking rhythmic with linear increase (*middle, upper left*), night peaking rhythmic with linear increase (*middle, upper right*), day peaking rhythmic with linear decrease (*middle, lower left*), night peaking rhythmic with linear decrease (*middle, lower right*), linear increase, not rhythmic (*bottom left*), and linear decrease, not rhythmic (*bottom right*). Data are plotted relative to DLMO, and by relative clock time, with relative bedtime at midnight. Errors bars represent SEM. The area between the dashed lines represent the ‘biological night’, defined as DLMO plus 10 hours, and the blue dashed line represents the predicted fit of the model.

We assessed the proportion of plasma metabolites in the targeted and untargeted matrices that changed in a rhythmic, linear or combined rhythmic and linear fashion over the 40-hours of extended wakefulness. Results of these analyses and model estimates for all statistical analyses are shown in SI Figs S1 and S2, and SI Tables 1–4 respectively. Representative examples of metabolites that exhibited rhythmic, linear and combined rhythmic and linear changes in plasma levels at the group-level are shown in Fig. 2B for the targeted matrix and in Fig. 3B for the untargeted matrix.

### Analysis of metabolites at the group-level

Group-level analysis of the 99 identified metabolites showed that 21 metabolites were significantly rhythmic and nearly all (90%) had a peak time (acrophase) during the biological night, occurring within 10 hours after Dim Light Melatonin Onset (DLMO). Furthermore, four metabolites showed a significant linear change; aconitic acid and uridine increased whereas phosphoric acid and proline decreased with time awake. In addition to the 21 rhythmic only and four linear only metabolites, seven of the 99 metabolites showed both rhythmic and linear changes. Five metabolites increased (threonine, cysteic acid, phenylalanine, ornithine and methylcitrate) and two decreased with time awake (pantothenic acid and lysine) and all had acrophases during the biological night. The 28 rhythmic metabolites (including combined rhythmic and linear metabolites), comprised 16 amino acids, 6 organic acids, 2 nucleotides, 2 carbohydrates and derivatives, and a single vitamin and peptide (see Table S1 for metabolite identities). Pathway analysis^29^ showed enrichment of the phenylalanine and tyrosine metabolism pathway (Table 2, and SI Fig. S3 and Table S6). The overall 11 linear metabolites (including combined rhythmic and linear metabolites), comprised 6 amino acids, 2 organic acids, and a single vitamin, xenobiotic and nucleoside (see Table S2 for metabolite identities). While the arginine and proline metabolism pathway showed enrichment, this was no longer significant following false discovery rate correction (SI Fig. S4 and Table S7). The time-course of all metabolites from the targeted matrix are shown in Fig. 2A.

**Table 2:** Results of the pathway enrichment analysis showing significant pathways*.

Analysis		Pathway	Total metabs in pathway	#sig. metabs in pathway	Raw p	FDR adjusted p
Group	Rhythmic	Phenylalanine and tyrosine metabolism	13	4	0.000623	0.0492
	Linear	—	—	—	—	—
Individual	Rhythmic	Citric acid cycle	23	9	0.000107	0.0085
		Urea cycle	20	8	0.000226	0.00892
	Linear	Citric acid cycle	23	10	2.41E-06	0.00019
		Urea cycle	20	8	5.83E-05	0.0023
		Malate-aspartate shuttle	8	4	0.00195	0.0337
		Beta-alanine metabolism	13	5	0.00207	0.0337
		Galactose metabolism	25	7	0.00213	0.0337

**Figure 4:**
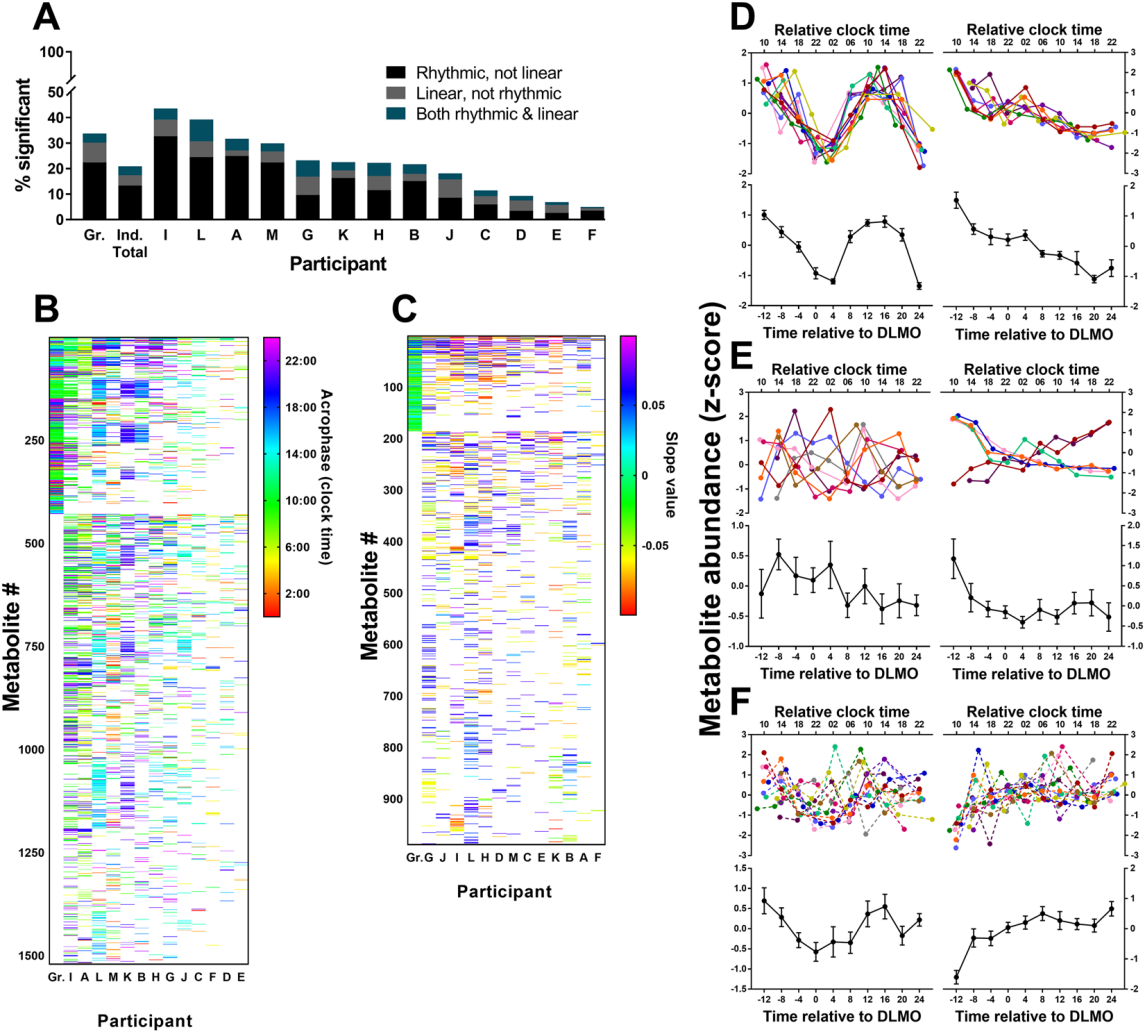
Comparison of group- versus individual-level analyses. (**A**) The percentage of metabolites that were significantly rhythmic (black), linear (grey), or combined rhythmic and linear (blue) at the group-level (Gr.), overall for the individual-level analysis (Ind. total), and for each individual participant (A–M). (**B**) Acrophase and (**C**) slope values are shown for significant metabolites (including combined rhythmic and linear metabolites) at the group-level (Gr.) and in individual participants (A–M). Metabolites are ordered based on the number of significant cosinor or linear fits across participants, with group-significant metabolites shown first. Participants are ordered from left to right based on the greatest number of significantly rhythmic or linear metabolites at the individual-level. (**D**) Metabolites that were significantly rhythmic (*left*) or linear (*right*) in group- and individual-level analyses. (**E**) Metabolites significantly rhythmic (*left*) or linear (*right*) in almost half of individuals but were not group-significant. (**F**) Metabolites that were significantly rhythmic (*left*) or linear (*right*) in group-level, but not individual-level analyses (non-significance denoted by broken lines in individual-level plots). Individual participant profiles are shown in colour and the group mean (±SEM) for that metabolite is shown below in black.

*Only metabolic pathways that were significant following FDR correction are shown. The total number of metabolites in the pathway, the number of significant metabolites in the pathway and the raw and FDR adjusted p-values are shown for each pathway. The full results of the pathway enrichment analysis are shown in SI Tables 6–9 and Figs 3–6.

Group-level analysis of the untargeted data showed a similar proportion of metabolites (~22%) to those in the targeted matrix were significantly rhythmic. A wide range in acrophase times were observed with many of the metabolites peaking during the daytime (60%). Group-level analysis of the untargeted matrix also showed that ~8% of metabolites were significantly linear, of which most (66%) of these decreased with time awake. Approximately 3% of metabolites showed a combined rhythmic and linear pattern of change. Just over half (51%) of these metabolites decreased over time and the majority (67%) of these peaked during the day. Of the metabolites showing combined rhythmic and linear trends that increased, the majority (72%) had an acrophase during the night. The time-course of metabolites from the untargeted matrix that were significantly rhythmic, linear or combined rhythmic and linear are shown in Fig. 3A.

### Analysis of metabolites at the individual-level

Following group-level analysis, we next analyzed individual participant metabolite profiles (i.e. single metabolite profiles over time for each participant), including the 1,287 (99 × 13 participants) targeted profiles and 21,333 (1,641 × 13 participants) profiles from the untargeted matrix. Results of these analyses are shown in SI Fig. 2. Of the profiles from the targeted matrix, ~10% were significantly rhythmic and over half (64%) of these rhythmic profiles peaked during the day. Profiles that were significantly linear accounted for ~5% of all analyzed profiles within the targeted matrix, and most (63%) of these showed an increase with time awake. Approximately 3% of individual profiles in the targeted matrix showed a combined linear and rhythmic pattern of change. Of these, over half (56%) increased with time awake, and 70% had acrophases during the night. In metabolites that decreased, however, there was an even spread of acrophases throughout the day and night.

The identified rhythmic compounds detected at the individual-level comprised mainly amino (29%) and organic acids (22%), although a number of nucleotides and nucleosides (16%) and carbohydrates and derivatives (14%) were also rhythmic. Amino acids had acrophases mainly during the evening and throughout the night. Similarly, organic acids predominantly peaked during the biological night, whereas carbohydrates and their derivatives had acrophases throughout the day and night. Nucleotides and nucleosides, however, tended to peak during the morning hours, in the first half of the day. Pathway analysis of these rhythmic compounds showed that the urea and Krebs cycle pathways were significantly enriched (see Table 2, and SI Fig. S5 and Table S8). Similar to the rhythmic metabolites, linearly changing metabolites comprised mainly amino acids (29%), organic acids (29%), carbohydrates (16%), and nucleotides and nucleosides (9%). The amino and carboxylic acids showed both increases (amino: 55%; carboxylic: 52%) and decreases (amino: 45%; carboxylic: 48%), whereas most of the carbohydrates increased (69%) and all nucleosides and nucleotides increased with time awake. Significantly enriched pathways for the linear metabolites included the Krebs and urea cycles, malate-aspartate shuttle, beta-alanine metabolism and galactose metabolism (see Table 2, and SI Fig. S6 and Table S9).

Of the 21,333 individual untargeted profiles, ~14% were significantly rhythmic and ~4% showed a linear change with time awake. Similar to the group-rhythmic metabolites, most (62%) of the significant individual-level rhythmic profiles peaked during the day. There was a near-even split in the direction of linear change, with just over half (54%) of metabolites decreasing with time awake. Of all the significant individual profiles, ~4% were combined rhythmic and linear. These profiles tended to show an increase (56%) with time awake and peaked mostly during the day (79% of those increasing, and 56% of those decreasing).

### Comparison of group- and individual-level analyses

We next examined the level of concordance in group- and individual-level analyses. As seen in Fig. 4A, the proportion of significant (rhythmic, linear or both rhythmic and linear) metabolites was decreased overall in the individual-level analysis (22%) compared to the group-level analysis (32%). This decrease appeared to be driven mainly by a reduction in significantly rhythmic metabolites; however, the proportion of linear metabolites also decreased slightly in the individual-level analysis of untargeted profiles. Figure 4 also shows the percentage of metabolites showing rhythmic (Fig. 4B, including combined rhythmic and linear metabolites) and linear (Fig. 4C, including combined rhythmic and linear metabolites) changes for each individual participant. The proportion of overall significant metabolites differed between participants, with some participants having less than 10% of metabolites showing rhythmic and linear changes during prolonged wakefulness (Fig. 4A).

As seen in Fig. 4B and 4C, there were differences between participants in which metabolites were significant, and there were no metabolites that were significantly rhythmic or linear for all participants. While not significant in all participants, there were metabolites that were relatively consistent across some participants (i.e. significant in at least n = 6), although, acrophase and slope estimates often differed substantially between participants for many of these metabolites. Due to this inter-individual variability, we observed a number of metabolite features, ~21% (6/28) and ~17% (4/23) for rhythmic and linear metabolites (including combined rhythmic and linear metabolites) that were significant in almost half of the individual participants profiles (n = 6/13), but not significant at the group-level. The observed inter-individual variability in acrophase also contributed to some group acrophase estimates not being representative of the timing of rhythms in individual participants, even when these metabolites were significant at both the group- and individual-level (Fig. 4B). For linear metabolites, however, group estimates of the direction of change generally reflected changes at the individual-level, although the magnitude of change was often decreased in the group estimate relative to individual participant profiles (Fig. 4C). Overall metabolites with consistent profiles between individuals were more likely to be significant at the group-level (Fig. 4D), and metabolites that had either a wide range in acrophases for rhythmic metabolites, or opposing slope directions for linear metabolites, were not significant at the group-level, despite being significant in approximately half of the participants (Fig. 4E). Surprisingly, we also observed a group of metabolites that were significant at the group-level but were not significant in a single individual, as seen in the examples shown in Fig. 4F. Approximately 6% (25/428) of group-rhythmic metabolite features and 15% (27/185) of group-linear metabolite features were not significant in a single participant. This proportion increased to ~65% for rhythmic and ~68% for linear metabolite features when all metabolites significant in less than a third of participants (n = 3/13) were included.

## Discussion

Our study presents the first evidence of circadian- and wake-dependent modulation of polar metabolites over the course of 40-hours of extended wakefulness. We describe rhythmic and linear changes in plasma metabolites at both the group- and individual- level. Due to large inter-individual differences observed in both circadian- and wake-dependent metabolites, our findings highlight the importance of data being examined at both the group-and individual-level for biomarker discovery work. For a biomarker discovery program, aiming to identify biomarkers of an abnormal state, the biomarker should ideally have utility at the individual-level. Targeting or rejecting a metabolite based on group-level data may therefore lead to inconclusive results or a missed signal of interest. With this in mind, while our data support earlier findings demonstrating rhythmic and/or linear changes in the human plasma metabolome during sleep deprivation^15,16,18–21^, they also suggest caution when interpreting results from analyses of grouped data.

Previous studies using reverse phase-LC-MS to detect changes in plasma metabolite levels, indicated that ~13–40% of the lipids and apolar metabolites preferentially detected by this platform are under circadian control^15,19–21^. We show that a similar proportion of polar metabolites detected using HILIC-LC-MS exhibited circadian rhythmicity in our group-level analyses (~22% or ~26% including metabolites that were combined rhythmic and linear). Furthermore, the timing of the peak of metabolite rhythms in our targeted matrix was consistent with previous reports that have shown amino acids to peak predominantly in the evening and during the biological night^15,20,21^. Ten (of 28, including those showing a combined rhythmic and linear pattern) metabolites found to be rhythmic in our targeted group of compounds were rhythmic in at least one other study^15,16,20,21^. These included leucine, lysine, methionine, valine, 4-hydroxyphenylpyruvate, isoleucine, tyrosine, ornithine, phenylalanine, and tryptophan. Furthermore, 4 metabolites (citrulline, arginine, citric acid and pantothenic acid) found to be rhythmic in the current study did not show rhythmicity in previous studies^15,16,20^ and 4 metabolites (proline, glutamate, lactate and glycerol) shown to be rhythmic in at least one previous study^16,20^ were not rhythmic in our dataset in group-level analyses but were rhythmic (except glutamate) in at least one participant. Differences in methodologies, for example CR versus non-CR conditions, meal timing and pooling samples, may account for the differences in results. The findings of the current study also confirmed lack of rhythmicity in 13 metabolites previously shown not to be rhythmic in at least one other study^16,20^ (e.g. taurine, uridine, serine, oxalate, pyruvate and AMP). Overall, our analyses using HILIC-LC-MS detected 12 previously unreported rhythmic metabolites, which included organic acids (e.g. methylcitric acid, fumaric acid, glucuronic acid, isocitric acid, and cysteic acid) and nucleotides (e.g. deoxyuridine monophosphate and inosinic acid).

Following our group-level analysis, we also analyzed individual participant profiles. These analyses showed that ~14% (~18% including combined rhythmic and linear metabolites) of individual participant metabolite profiles were rhythmic. Similarly, despite the difference in the classes of measured metabolites, Chua *et al*.^19^ reported that ~18% of lipid metabolite profiles were rhythmic, suggesting that a similar proportion of lipid and polar metabolites are under circadian control. Based on the identified metabolites from the targeted matrix, the rhythmic metabolites in the current study were predominantly amino and organic acids, such that pathways involving these classes of metabolites, including the Krebs and urea cycles, showed enrichment. While some metabolites involved in the urea and Krebs cycles had acrophases during the biological night, most had acrophases during the day, consistent with the diurnal peak in urea concentration^30,31^ and energy expenditure reported in the literature^32^. Furthermore, the timing of amino acid rhythms in the current study is broadly consistent with previous research showing that transcripts associated with gene expression and RNA metabolism tend to peak during the night^33–35^, such that that the timing of amino acids observed in the current study coincides with the timing of protein synthesis.

As has been reported previously for plasma lipids^19^, none of the polar metabolites were consistently rhythmic across all individuals and we observed a large degree of inter-individual variation in the timing of rhythms between participants. As seen in Fig. 3B, some participants appeared to have a similar timing of rhythms across most metabolites (e.g. acrophase estimates for participant K were mostly during the evening hours), suggesting that some individuals may have a particular phase predominance in their metabolic profile. A similar finding was observed in plasma lipids, whereby participants could be clustered into morning and evening phenotypes based on the peak times of lipid rhythms^19^. Further characterization of the range of inter-individual variability in metabolites within and between individuals is necessary if these are to be used as potential biomarkers. The wide range of individual phases observed in metabolites is not surprising, however, when the inter-individual variation in well-established circadian markers is taken into consideration. Melatonin, the gold standard marker of circadian phase, when measured under dim light conditions exhibits an ~5-h range of phase (5.85-h in the current sample) and phase angles (DLMO time relative to sleep) in young healthy individuals (e.g.^36,37^). Even within individuals, there is also variation in internal phase relationships, for example, between melatonin and temperature^38^, not only because of methodological variance but also likely intrinsic differences in internal circadian organization. It is therefore important to interpret potential new circadian markers with similar expectations, i.e., that substantial inter-individual variation will exist, and biomarkers are not likely to exhibit identical timing between, or even within individuals, but that does not preclude their use as circadian biomarkers. A long-term goal of this work is to better understand the inter-individual variation in biomarker profiles to inform the eventual development of single- or dual-timepoint markers of circadian phase for clinical and operational use, as has already been attempted using both the human metabolome^21^ and transcriptome^39,40^.

Given large inter-individual differences in metabolites, we sought to compare the results of our group- and individual-level analyses. Overall, metabolites that had consistent profiles between participants tended to be significant at the group-level, while those with a large spread of acrophases were typically not significant at the group-level. Furthermore, we observed that 25 of 428 metabolites were significant at the group-level despite not being significant in a single individual. These findings are important given the widespread use of group-level analyses in previous studies assessing circadian rhythms in the metabolome^15,16,20,41^.

In addition to identifying rhythmic metabolites, we also identified approximately 8% (11% including combined rhythmic and linear metabolites) of metabolites that showed a linear increase or decrease with time awake in group-level analyses. The proportion of linearly changing metabolites in the current study is similar to the proportion of metabolites that showed an increase or decrease in response to acute sleep deprivation (~12%^20^), and sleep restriction to 5.5-h time-in-bed for 8 nights (~4%^42^). Despite similarity in the proportion of metabolites increasing or decreasing, metabolites that changed linearly in the current study are not consistent with the metabolites that changed in response to chronic (5–8 nights) sleep restriction^42,43^, such that a number of metabolites that were altered by sleep restriction did not show a wake-dependent change in the present study (e.g methionine, tryptophan, oxalic acid, gluconic acid, malic acid and glucose). Furthermore, 3 metabolites showing a linear change in the current study did not change in response to chronic sleep restriction^42,43^ (cis-aconitic acid, lysine and threonine). Phenylalanine, however, which showed an increase with extended wakefulness in the current study also showed an increase following 5 nights of 4-h time-in-bed^43^ but did not change following 8-nights of 5.5-h time-in-bed^42^. The difference suggests that biomarkers signaling sleep loss due to acute sleep deprivation may not be the same as those sensitive to chronic sleep deficiency, which is consistent with that reported using a transcriptomic approach^44^. Another possible explanation is that the 24-hour rhythm of some metabolites was shifted due to the sleep restriction protocol (as seen for melatonin^45,46^), such that the change attributed to sleep restriction may represent measurement at a different phase of the rhythm. This may be the case for some of the metabolites identified as markers of sleep restriction, for example tryptophan, phenylalanine, and isoleucine, as these metabolites have been shown to be rhythmic, both in the current study and in previous research^15,16,21^. Further investigation is required to determine whether the metabolites that show wake-dependent increases or decreases in response to sleep deprivation are also altered by sleep restriction, or whether there are different mechanisms resulting in a different set of metabolites showing change in response to the sleep deprivation versus sleep restriction.

Our data examining wake-dependent changes in metabolites during acute sleep deprivation largely confirm those reported previously^16,20^ with respect to the metabolites which did not change in response to sleep loss. For example, 23 metabolites that showed no linear change in the current study also did not change in response to sleep deprivation in previous studies^16,20^. We did, however, detect a linear change in 8 metabolites which were found not to change in response to sleep deprivation in previous research (phenylalanine, pantothenic acid, ornithine, uridine, threonine, proline, lysine and cis-aconitic acid). Furthermore, our results differed to previous studies showing increases in lactid acid^20^, taurine and tryptophan^16^ (though tryptophan did not change in one previous study^20^) as we did not show a linear change with time awake in group-level analyses for these metabolites. With the use of HILIC-LC-MS in our study however we were able to detect linear changes in 3 metabolites (phosphoric acid, cysteic acid, 2-methylcitric acid) not previously captured in other studies.

In our analysis of individual participant metabolite profiles, we found that 4% (~8% including combined rhythmic and linear metabolites) of metabolite profiles changed linearly. Based on the targeted analyses, these were mainly amino and organic acids, as well as a smaller number of carbohydrates. Enrichment analysis showed that the linearly changing metabolites were related to energy metabolism in the glycolysis and Krebs cycle pathways. While some of the metabolites in these pathways decreased, the majority increased with time awake and this is consistent with the reported increase in energy expenditure during sleep deprivation^32,47^. The urea cycle pathway also showed enrichment, with majority of the metabolites involved in this pathway showing an increase with time awake, which is consistent with the increase in urea in response to sleep loss^30,48^.

Comparable to the rhythmic metabolites, there was also inter-individual variation in the patterns of change of linear metabolites, such that the magnitude, and in some cases the direction of change, differed between participants (Fig. 4C). While these different responses between participants may indicate differential vulnerability to the metabolic consequences of sleep loss, confirmation requires further investigation to determine whether these inter-individual responses to sleep loss are stable and trait-like. Our finding of inter-individual variation in linearly changing metabolites is consistent with the large inter-individual differences reported in lipids showing wake-dependent changes^18^, although our results suggest that polar metabolites are less likely to change with time awake than lipid species (27% lipid *vs* 8% polar). As with our analysis of rhythmic metabolites, we observed differences in which metabolites showed significant linear changes depending on whether the data were analysed at the group- or individual-level. For example, we observed that 29 of the 188 metabolites detected as significantly linear at the group-level were not significantly linear in a single participant. This discrepancy highlights the importance of using both group- and individual-level analyses in biomarker discovery, as had we only conducted a group-level analysis, significant resources may have been used in trying to identify and validate group-significant metabolites that lack utility as a biomarker at the individual-level. There were a small number of unidentified linear metabolites from the untargeted matrix, however, that showed strikingly consistent changes across majority of the participants (e.g. right panel of Fig. 4D). Metabolites such as these may be useful as biomarkers of sleep pressure, however, further work to identify these metabolites and to validate our results is required.

Our study has three main strengths. First, our data are novel in that our analytical approaches allowed detection of a broad range of polar and non-polar metabolites, extending the range of metabolites that had previously been detected. While circadian- and wake-dependent changes have been previously described in moderately polar metabolites under CR conditions^20,21^, our findings, showing large inter-individual differences in the circadian phase of polar metabolites, suggest that these prior data were potentially confounded by the pooling of samples from multiple participants at the same clock time. Second, our study is the first to employ both group- and individual-level analyses to examine 24-hour rhythms and wake-dependent changes in polar metabolites. Differences between group- and individual-level analyses have only been investigated in plasma lipids^18,19^, while other studies that have measured both moderately and non-polar compounds have reported data at the group-level^15,16,20,42,43^. Finally, our sample includes female participants, which have not been included in some previous publications (e.g.^15,16,18–21^). While the small number of females precludes extensive analyses of sex differences, we did observe that four metabolite features were significantly rhythmic in 3 of 4 women and no men, and a further four were significantly linear in 3 of 4 women and no men. While these preliminary findings suggest there may be sex differences in the expression of some metabolites, further research with larger numbers of men and women, and equal group sizes is required to further elucidate possible sex differences in circadian and wake-dependent changes in plasma metabolites.

This study comprises the first step within a larger biomarker discovery program, where the current study was designed to produce proof-of-concept data within a small, but highly controlled study. The small sample size or the frequency of sampling, however, means that the current study may have been underpowered to detect some rhythmic or linear changes at the group-level, particularly those with low amplitudes or shallow slopes. Despite this, our sample size and sampling frequency is commensurate to previous metabolomics studies^15,16,20^. Future validation studies should be conducted on larger populations with more frequent sampling (e.g. 2-hourly). Furthermore, as with previous studies, participants in the current study were all young and extremely healthy, and the laboratory conditions were highly controlled during the CR protocol. While at this stage in the biomarker development process it is important to first identify the presence of any circadian and wake-dependent changes in an homogenous sample under highly controlled conditions, these findings will need to be validated in other populations and in less controlled, applied settings including circadian misalignment, sleep restriction, and in field settings. To this end, future inclusion of a control group with a normal sleep/wake schedule, ambulatory activity and typical food intake would aid in determining whether any linear changes observed were due to external factors. Finally, while we identified metabolites that changed linearly during sleep deprivation it is difficult to ascertain whether these metabolites are directly under the control of the sleep homeostat and are truly wake-dependent, or perhaps represent something else, for example, a build-up of certain metabolites from the hourly meals given during the CR. Similarly, the use of a CR protocol makes it difficult to uncouple the contribution of the circadian system and the sleep homeostat. Future studies might employ a forced desynchrony protocol to allow for a more comprehensive investigation of the individual contribution of the homeostatic and circadian processes on the abundance of specific metabolites.

To our knowledge this is the first study to characterize circadian- and wake-dependent changes in polar plasma metabolites. Our results describe circadian- and wake-dependent control of the polar metabolome and highlight the importance of analyzing these types of data at both the group- and individual-level. We showed that analysis at the group-level resulted in inaccurate measures of the abundance and time-course of both rhythmic and linearly changing metabolites. Underlying inter-individual differences in circadian- and wake-dependent modulation of the metabolome will also likely be an important consideration for future biomarker development programs using metabolomics.

## Methods

### Participants

Fourteen healthy adults (13 following exclusion of n = 1, 4 females, 24.74 ± 4.09 years; Table 1) completed a 6-day in-laboratory study. Participants were free from medical, psychiatric or sleep disorders, had not engaged in night- and/or shift-work in the past three years, or travelled across more than one time zone in the past three months. Two weeks prior to the laboratory study, participants maintained a self-selected 8:16 sleep-wake schedule, which was confirmed by wrist actigraphy (Actiwatch Spectrum, MiniMitter Inc, Bend, OR) and sleep diaries. The use of prescription and over-the-counter medications, supplements, recreational drugs (also exclusionary if consumed in the previous month based on self-report), nicotine, caffeine, and alcohol were not permitted from 3 weeks prior to admission until completion of the study. Urine drug screening, and a pregnancy test for women, was conducted prior to laboratory admission. All participants provided informed consent and the study was approved by the Monash University Human Research Ethics Committee (CF14/2790 – 2014001546). The experiments were conducted in accordance with the Declaration of Helsinki.

### Study Protocol

Participants were continuously monitored for 6-days in an environment free of time cues. There was no access to windows, clocks, live TV, or newspapers, and participants were supervised by technicians trained not to reveal time of day. Women were studied during their follicular phase, with admit occurring immediately after their last menses, to minimize differences between women due to menstrual phase. The study started with two baseline nights with sleep scheduled at the same time as participants’ self-selected sleep in the two weeks prior to admission. Full polysomnography was recorded on the first night to confirm no presence of sleep disorders, including restless legs syndrome, periodic limb movements, and sleep disordered breathing. During baseline days, participants were fed three main meals and three snacks per day.

Upon waking on Day 3, participants commenced a 40-h constant routine (CR). During the CR, participants remained awake under constant supervision in dim light conditions (<3 lux), in a semi-recumbent posture (head of bed at 45°), and received identical hourly snacks (quarter sandwich, 60 ml water and 40 ml apple juice). The calorie content of hourly snacks was ~1.1 x the average resting energy expenditure (REE) for all participants (1796 ± 236 cal/day) and the macronutrient content adhered to the recommendations of the Australian Dietary Guidelines 2013^49^. REE for each participant was calculated as^50^:

$$RE{E}_{Male}=9.99\ast weight(kg)+6.25\ast height(cm)-4.29\ast age+5$$

$$RE{E}_{Female}=9.99\ast weight(kg)+6.25\ast height(cm)-4.29\ast age-161$$

Participants had the choice of four sandwich options that were approximately equivalent in calorie (1804 ± 99 cal/day) and macronutrient content (~20% protein, ~33% fat, ~46% carbohydrate). Each participant received only one of the sandwich options for the duration of the CR.

### Lighting

During baseline and recovery days, maximum ambient light during wake episodes was ~100.9 ± 18.2 lux when measured in the horizontal plane and ~44.0 ± 13.9 lux when measured in the vertical plane at the height of 182 cm. On baseline night 2 and during CR, lights were dimmed to ~2.8 ± 0.5 lux in the horizontal plane and ~1.2 ± 0.3 lux in the vertical plane when measured at 182 cm. During scheduled sleep episodes, ambient lighting was turned off. The room lighting was generated from ceiling-mounted 4100 K fluorescent lamps (Master TL5 HE 28W/840 cool lights, Philips Lighting, Amsterdam, Netherlands) that were covered with neutral density filters (3-stop LEE Filters, Lightmoves, Noble Park, Australia). Illuminance measures (J17 Lumacolor photometer, Tektronix, Beavertown, USA) were taken daily in four locations around the room, positioned directly under light panels.

### Blood sample collection and processing

Plasma samples were collected during the CR via an indwelling intravenous cannula, inserted into the forearm or antecubital vein approximately 1-hour after wake. Blood was collected hourly for plasma melatonin assay, and additional blood was collected every 2-hours for metabolomics analysis starting 2-hours post wake. At each collection, whole blood was collected in a syringe and aliquoted into a blood tube spray coated with K2EDTA. Samples were immediately centrifuged at 4 °C, or stored in a fridge at 4 °C for up to ~30 minutes until processing. Samples were spun at 1,300 × g for 10 minutes and plasma was aliquoted into 500 µL fractions and temporally stored on dry ice before transfer to permanent storage at −80 °C within 4–12 hours.

Of the total 546 scheduled blood collections (39 samples × 14 participants), 529 were collected successfully (3.11% missing samples) and assayed for melatonin. For the metabolomics analysis, up to ten 4-hourly samples per participant were analyzed at times 2, 6, 10, 14, 18, 22, 26, 30, 34 and 38 hours post-wake. Of the 140 possible samples, 18 (13.6%) were missing due to either a missed collection (n = 1 sample) or had moderate to severe haemolysis (orange to pink in colour; n = 17 samples). To be included in the metabolomics analysis, participants could not have more than 70% missing blood samples, and no more than two consecutive missing samples. To avoid excluding two individuals, a 4-hourly sample was replaced with a successful collection occurring 2 hours before or after the sample that required replacement— for example, a sample collected at 32 hours was used to replace a missing sample at 34 hours. With these replacements, a total of 124 samples were included in the final metabolomics analysis from 14 participants.

Of the 124 plasma samples analysed using LC-MS, five samples were lost in both the targeted and untargeted matrices post-analysis due to mis-injection into the LC-MS (n = 119 samples). Retention time drifts (>2 mins) resulted in the exclusion of an additional three samples from the untargeted matrix following XCMS analysis (n = 116), but not from the targeted matrix which was manually integrated such that the retention time window could be widened to incorporate these metabolites (n = 119). One male participant was excluded entirely from further analysis, as this additional loss of samples resulted in three consecutive missing time-points in the middle of their data series making it difficult to interpret the model fits. Following removal of this participant, 10 of 13 participants had missing data points in the targeted matrix resulting in 14% (18 samples; total n = 112) missing data, and 12 of 13 participants had missing data points in the untargeted matrix resulting in a total of 16% (21 samples; total n = 109) missing data. Demographic information of the 13 participants included in the final analysis are shown in Table 1.

### Marker of the circadian clock

Total blood plasma melatonin was determined at the Adelaide Research Assay Facility (ARAF; University of South Australia, Adelaide, Australia) by reverse-phase C-18 column extraction of 500 µl plasma, followed by double antibody radioimmunoassay using standards and reagents supplied by Buhlmann Laboratories (RKMEL-2, Buhlmann Laboratories AG, Schönenbuch, Switzerland). The sensitivity of the assay using 500 µl of extracted plasma was 1.0 pg/ml. Samples were assayed in duplicate and the intra-assay coefficient of variation of the assays was 7.61%. The inter-assay coefficient of variation of the low concentration quality control was 11.03%, and the inter-assay coefficient of variation of the high concentration quality control was 13.08%.

To determine circadian phase, Dim Light Melatonin Onset (DLMO) was defined as the time at which plasma melatonin levels reached 5 pg/ml in the first cycle of the CR, calculated by interpolating between two adjacent samples^51^. For two participants, DLMO was calculated from the second cycle due to missing samples in the first 24-h cycle. The biological night was defined as DLMO plus 10 hours (DLMO + 10) and split according to first half (first 5 hours) and second half (second 5 hours) of the night. The biological day was defined hours the 14 hours between DLMO + 10 and DLMO. The biological day was further broken down into two equal 7-h halves.

### Metabolomics analysis

Metabolomics analysis was performed on plasma samples collected at 4-hourly intervals starting 2-hours post wake (Metabolomics Australia, Bio21 Molecular Science and Biotechnology Institute, University of Melbourne, Parkville, Australia). Samples were thawed on ice and 20 µL of plasma was aliquoted for analysis by LC-MS. An additional 20 µL from each sample was pooled to generate a plasma quality control (PQC) sample, from which aliquots were taken in preparation for extraction with plasma samples. Plasma samples and PQCs were extracted using 180 µL acetonitrile/methanol (1:1 v/v) solution containing 2 µM ^13^C-sorbitol, 2 µM ^13^C^15^N-AMP, and 2 µM ^13^C^15^N-UMP as internal standards. Samples were vortexed for 30 seconds, sonicated for 5 minutes at 4 °C, then incubated for 10 minutes at 4 °C (in an Eppendorf Thermomixer). Sample (prepared in batches of 24, with a PQC every 10 samples) were centrifuged (4,500 × g, 10 minutes, 4 °C) and 180uL of the supernatant was transferred into a glass vial. An aliquot of each sample of the extracts (10 µL) was pooled to create a pooled biological quality control (PBQC) sample.

Samples (10 µL) were resolved on a ZIC®-pHILIC column (5 µm particle size, 150 × 4.6 mm, Merck SeQuant®) connected to an Agilent 1260 (Santa Clara, CA, USA) HPLC system running a 29.5-minute gradient with mobile phases 20 mM ammonium carbonate (pH 9.0; Sigma-Aldrich; Solvent A) and 100% acetonitrile (solvent B) at a constant flow rate of 300 µL/min. The elution gradient started at a composition of 80% solvent B and decreased to 30% solvent B in 18.5-minutes for 6.5-minutes. Extracted plasma volumes of 7 µL were injected onto the column (maintained at 25 °C). Metabolites were detected by electrospray ionization using an Agilent 6545 Q-ToF MS system (Santa Clara, CA, USA) in negative ionization mode. The instrument was cleaned and calibrated weekly to ensure a mass accuracy of ±0.2 ppm. Detailed Q-Tof MS parameters can be found in Stewart, *et al*.^52^. Samples were analysed in the same analytical batch and randomized by participant and time, with a QC every 5 samples. PQCs were run every tenth sample to monitor any batch preparation effects and PBQCs were run every tenth sample to monitor instrument performance during the run. Solvent blanks were analysed every 24 samples to monitor background. Five mixtures of authentic standards (234 metabolites) were also run to generate a library for the targeted analysis.

Metabolite identification for the targeted analysis (targeted matrix) was based on accurate mass, retention time and MS/MS fragmentation patterns for metabolites in the standard mixtures. Relative abundances based on area under the metabolite peak were obtained using MassHunter Quantitative Analysis B 0.7.00 (Agilent). Metabolites with low quality chromatographic peaks (<10,000 area count) and peaks not reliably detected across samples were excluded resulting in the detection of 99 (of 234) metabolites [Level 1 confidence according to the Metabolomics Standard Initiative^53^]. The untargeted matrix containing 1,641 metabolite features, which include metabolite features already represented in the targeted matrix, was generated by XCMS *centWave* algorithm^54^ to detect molecular features in the raw files and the features list was further refined in CAMERA^55^ to group related features by annotating isotope and adduct peaks.

### Statistical analysis

To reduce biological variability between participants and timepoints, raw area count data were normalized relative to the median metabolite abundance for each individual sample. Given differences in the relative concentration of metabolites between individuals, the median normalized data were z-scored in order to scale the data prior to analysis. Data were z-scored relative to the mean and standard deviation of each participants’ scores for a single metabolite. Each time-point was then expressed relative to DLMO, where DLMO was defined as time 0. Grouped data were averaged across 4-hour phase bins to align the data points relative to each participant’s internal circadian time. Data were fitted with a non-linear regression model^18^ that was comprised of a cosinor function with a linear component:

$$y=A\,\cos \{2\pi (\frac{t-\varphi }{\tau })\}+Ct+D$$

In the model, *A* is the amplitude of the sinusoid, *τ* is the period set at 24-h, *t* is time, *Ø* is the acrophase of the sinusoid, and *C* and *D* are the slope and y-intercept of the linear component, respectively. Fitting of the model was conducted in SAS 9.4 using the *proc nlin* procedure. The model was fitted to the 99 and 1,641 metabolite profiles averaged within phase bins from the targeted and untargeted matrix (group-level analysis), and to each individual participants’ metabolite profiles from the targeted and untargeted matrix (individual-level analysis). The cosinor and linear components of the regression were considered significant if the amplitude and slope, respectively, were significantly different from 0. Where the regression model detected a significant nadir, acrophase was calculated as the peak 12-hours later. Given the exploratory nature of the study, p-values were set at 0.05. The model estimates for all analyses, including amplitude, acrophase and slope estimates are shown in SI Tables 1–4.

Pathway enrichment analysis was conducted in MetaboAnalyst 3.0 (http://www.metaboanalyst.ca) using the Enrichment Analysis module. This analysis provides a p-value for the overall likelihood that a metabolite set or pathway is involved based on the metabolites entered, and also indicates the degree of enrichment (fold enrichment), which is representative of how many metabolites within a specific pathway are present in the metabolite set entered into the analysis. For example, if two out of four metabolites in a pathway are present then that pathway will show a greater fold enrichment than a pathway that has two out of 10 metabolites that are present. Pathway enrichment analysis was conducted separately for linear and rhythmic metabolites at the group and individual level. Metabolites that were combined rhythmic and linear were included in both analyses. The results of the pathway enrichment analyses generated in MetaboAnalyst 3.0 are shown in SI Figs 3–6 and SI Tables 6–9.

## Supplementary information

Supplemental information

Supplemental dataset 1

## Data Availability

Raw area count data from the untargeted matrix, which includes metabolite features in the targeted matrix, is available as supplementary information (Table S5). Requests for further data access will be considered on a case-by-case basis. Applications for data access should be sent to Dr. Clare Anderson (clare.anderson@monash.edu).
