# Supplementary material for: Circadian and wake-dependent changes in human plasma polar metabolites during prolonged wakefulness: A preliminary analysis
Source: Sci Rep. 2019 Mar 14;9:4428. doi: 10.1038/s41598-019-40353-8 (PMC6418225; doi:10.1038/s41598-019-40353-8)
Supplement: Supplementary file 1 — Supplemental information [file 41598_2019_40353_MOESM1_ESM.pdf]

Supplementary information to Grant et al.

Circadian and wake-dependent changes in human plasma polar metabolites during prolonged wakefulness: A preliminary analysis

Leilah K. Grant, Suzanne Ftouni, Brunda Nijagal, David P. De Souza, Dedreia Tull, Malcolm J. McConville, Shantha M. W. Rajaratnam, Steven W. Lockley, Clare Anderson

The following four tables are provided in xlsx format.

Table S1. Model estimates for rhythmic metabolites from the targeted matrix. The p-value, amplitude (Am) and acrophase (AcPh) for significant metabolites are shown for individual participants (A-M), and group-level analyses. Metabolite names and classes are given in the first two columns. The number of participants in which a single metabolite is significant is shown in column AP. Metabolites shown in bold demonstrated a combined rhythmic and linear pattern of change.

Table S2. Model estimates for linear metabolites from the targeted matrix. The p-value and slope for significant metabolites are shown for individual participants (A-M), and group-level analyses. Metabolite names and classes are given in the first two columns. The number of participants in which a single metabolite is significant is shown in column AC. Metabolites shown in bold demonstrated a combined rhythmic and linear pattern of change.

Table S3. Model estimates for rhythmic metabolites from the untargeted matrix. The p-value, amplitude (Am) and acrophase (AcPh) for significant metabolites are shown for individual participants (A-M), and group-level analyses. The number of participants in which a single metabolite is significant is shown in column AO. Metabolites shown in bold demonstrated a combined rhythmic and linear pattern of change.

Table S4. Model estimates for linear metabolites from the untargeted matrix. The p-value and slope for significant metabolites are shown for individual participants (A-M), and group-level analyses. The number of participants in which a single metabolite is significant is shown in column AB. Metabolites shown in bold demonstrated a combined rhythmic and linear pattern of change.

Table S5. Raw area count data from the untargeted matrix, which includes metabolite features represented in the targeted matrix, for all participants (A-M, and participant N excluded from primary analysis) at times 02-38 hours since wake, PBQCs and PQCs.

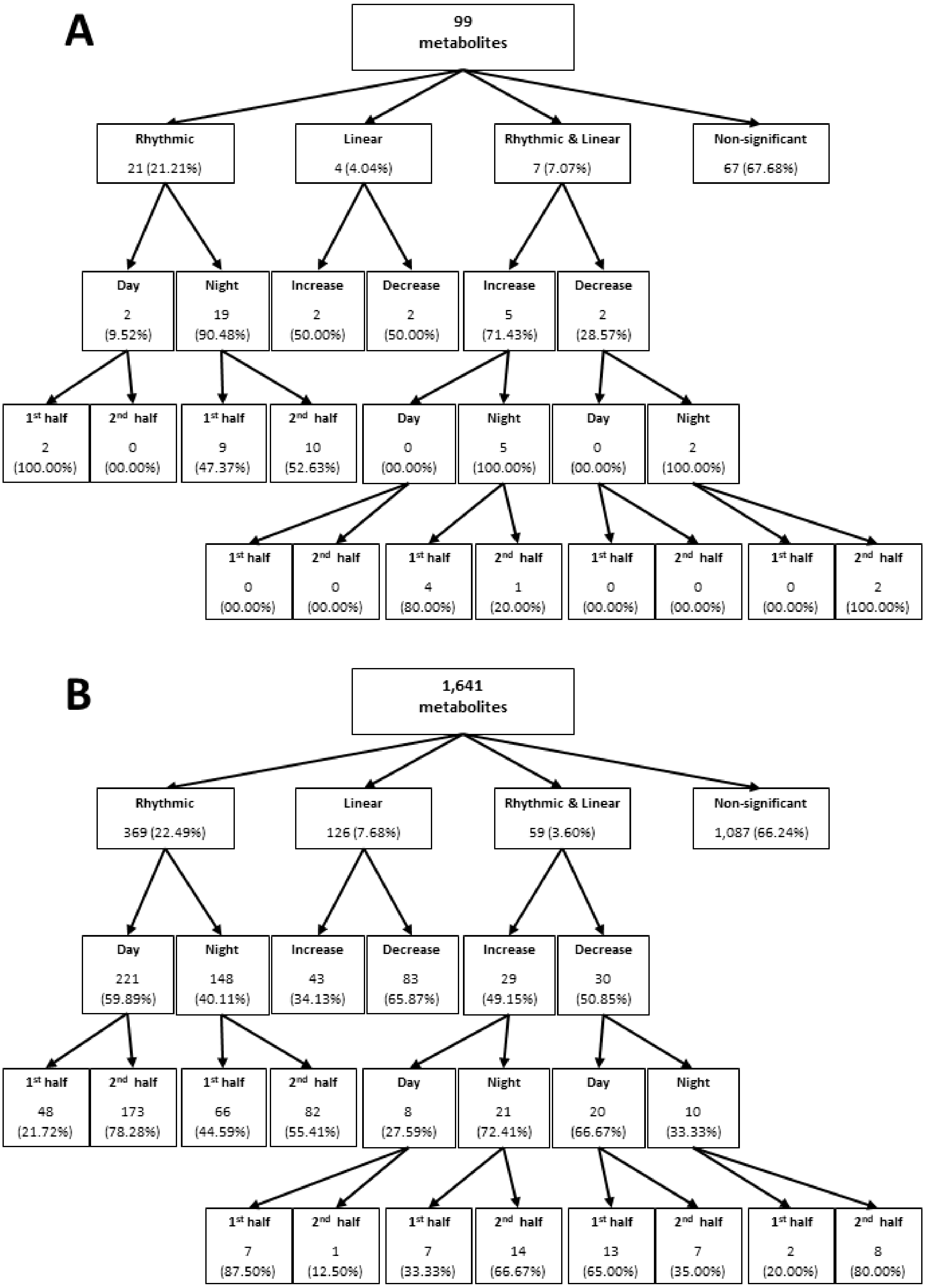

Figure S1. Results of the group-level analyses for the (**A**) targeted and (**B**) untargeted matrices showing the proportion of rhythmic, linear, combined rhythmic and linear, and non-significant metabolites. Rhythmic metabolites are further classified by acrophase (day/night and $1^{st}/2^{nd}$ half of day or night), and linear metabolites are classified according to the direction of linear change (increasing or decreasing).

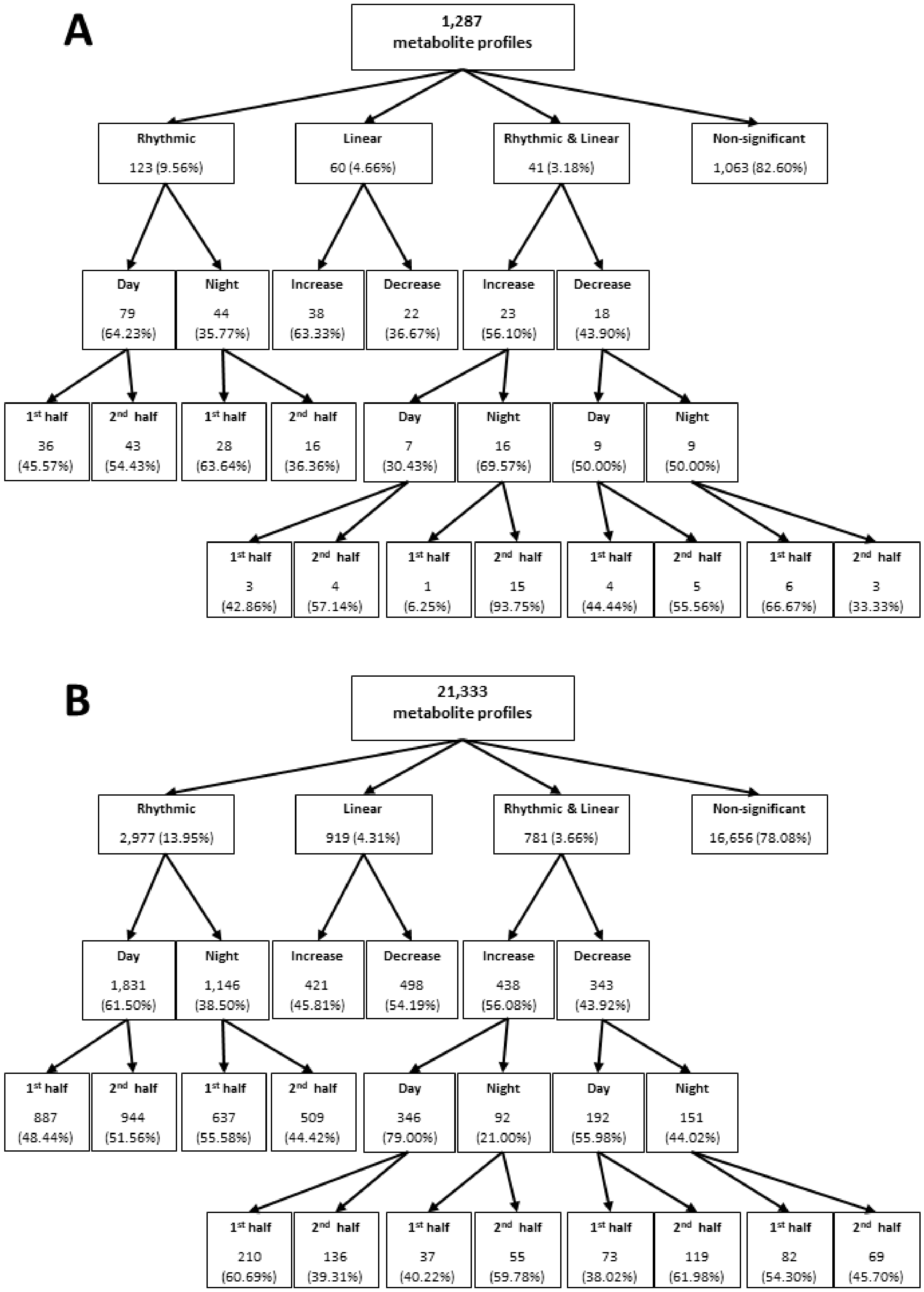

Figure S2. Results of the individual-level analyses for the (**A)** targeted and (**B**) untargeted matrices showing the proportion of rhythmic, linear, combined rhythmic and linear, and non-significant metabolite profiles. Rhythmic metabolites are further classified by acrophase (day/night and 1st/2nd half of day or night), and linear metabolites are classified according to the direction of linear change (increasing or decreasing).

Table S6. Results of the pathway enrichment analysis of identified group-rhythmic (including combined rhythmic and linear) metabolites from the targeted matrix. The table shows the pathway involved, the total number of metabolites in that pathway, the number of significantly rhythmic metabolites in that pathway and the raw and false discovery rate (FDR) adjusted p values. Results were generated from MetaboAnalyst 3.0.

Pathway	Total metabs. in pathway	# sig. metabs. in pathway	Raw p	FDR adjusted p
Phenylalanine and tyrosine metabolism	13	4	0.000623	0.0492
Urea cycle	20	4	0.00358	0.141
Aspartate metabolism	12	3	0.00633	0.167
Arginine and proline metabolism	26	4	0.00957	0.189
Citric acid cycle	23	3	0.0396	0.625
Starch and sucrose metabolism	14	2	0.0789	1
Valine, leucine and isoleucine degradation	36	3	0.119	1
Biotin metabolism	4	1	0.13	1
Inositol metabolism	19	2	0.134	1
Tyrosine metabolism	38	3	0.134	1
Catecholamine biosynthesis	5	1	0.159	1
Methionine metabolism	24	2	0.195	1
Taurine and hypotaurine metabolism	7	1	0.216	1
Nucleotide sugars metabolism	9	1	0.269	1
Pantothenate and CoA biosynthesis	10	1	0.294	1
Betaine metabolism	10	1	0.294	1
Beta-alanine metabolism	13	1	0.365	1
Lysine degradation	13	1	0.365	1
Mitochondrial electron transport chain	15	1	0.408	1
Ammonia recycling	18	1	0.467	1
Propanoate metabolism	18	1	0.467	1
Pentose phosphate pathway	18	1	0.467	1
Glycolysis	21	1	0.521	1
Galactose metabolism	25	1	0.585	1
Glycine, serine and threonine metabolism	26	1	0.599	1
Gluconeogenesis	27	1	0.613	1
Tryptophan metabolism	34	1	0.699	1
Pyrimidine metabolism	36	1	0.72	1
Purine metabolism	45	1	0.798	1

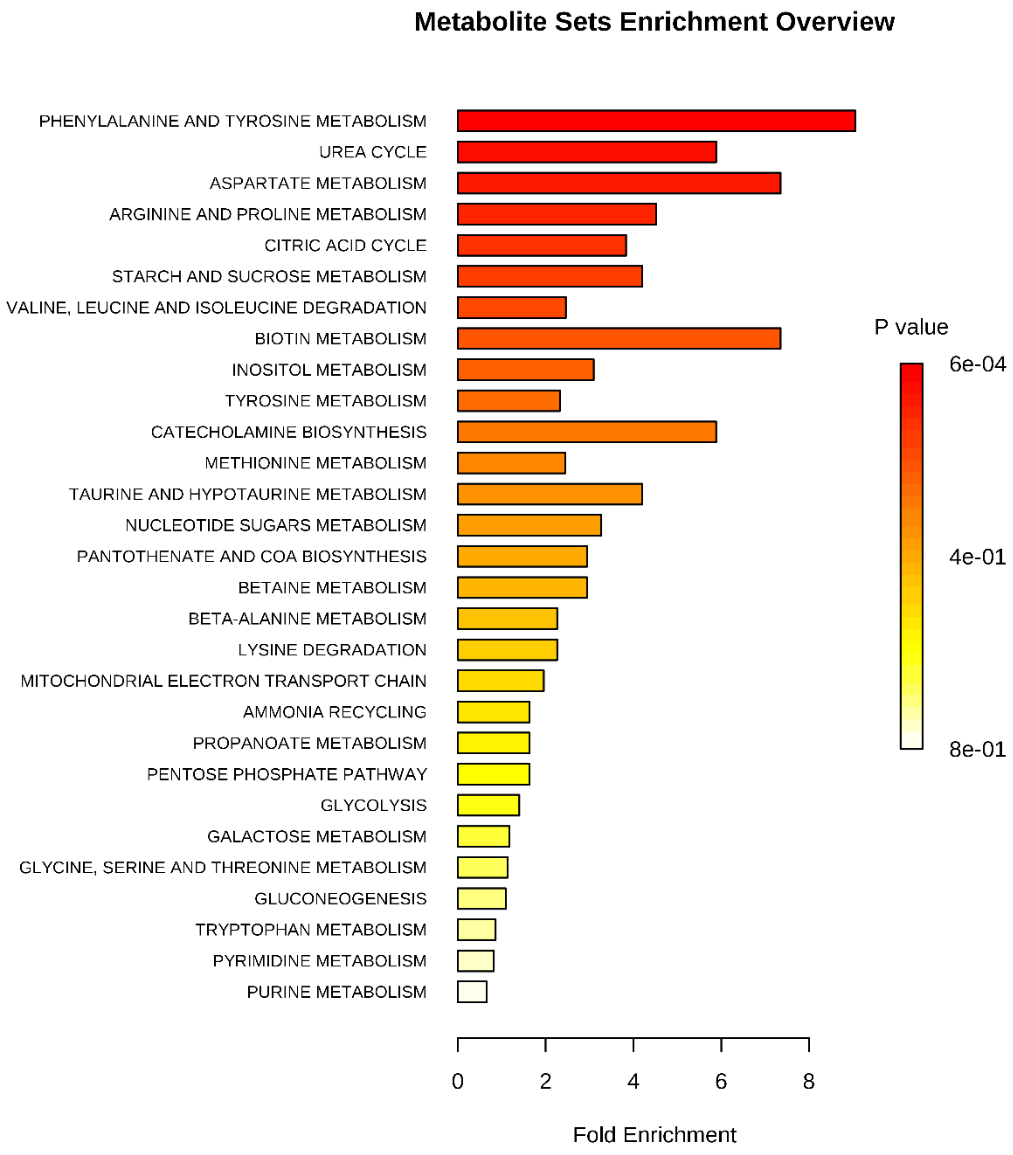

Figure S3. Pathway enrichment analysis of metabolites that were significantly rhythmic (including combined rhythmic and linear metabolites) in group-level analyses. Unadjusted p-values are represented. Generated by MetaboAnalyst 3.0.

Table S7. Results of the pathway enrichment analysis of identified group-linear (including combined rhythmic and linear) metabolites from the targeted matrix. The table shows the pathway involved, the total number of metabolites in that pathway, the number of significantly linear metabolites in that pathway and the raw and false discovery rate (FDR) adjusted p values. Results were generated from MetaboAnalyst 3.0.

**Pathway**	**Total metabs. in pathway**	**# sig. metabs. in pathway**	**Raw p**	**FDR adjusted p**
Arginine and proline metabolism	26	2	0.0443	1
Biotin metabolism	4	1	0.0525	1
Taurine and hypotaurine metabolism	7	1	0.0902	1
Pantothenate and CoA biosynthesis	10	1	0.127	1
Beta-alanine metabolism	13	1	0.162	1
Phenylalanine and tyrosine metabolism	13	1	0.162	1
Lysine degradation	13	1	0.162	1
Urea cycle	20	1	0.238	1
Citric acid cycle	23	1	0.269	1
Glycine, serine and threonine metabolism	26	1	0.299	1
Pyrimidine metabolism	36	1	0.39	1

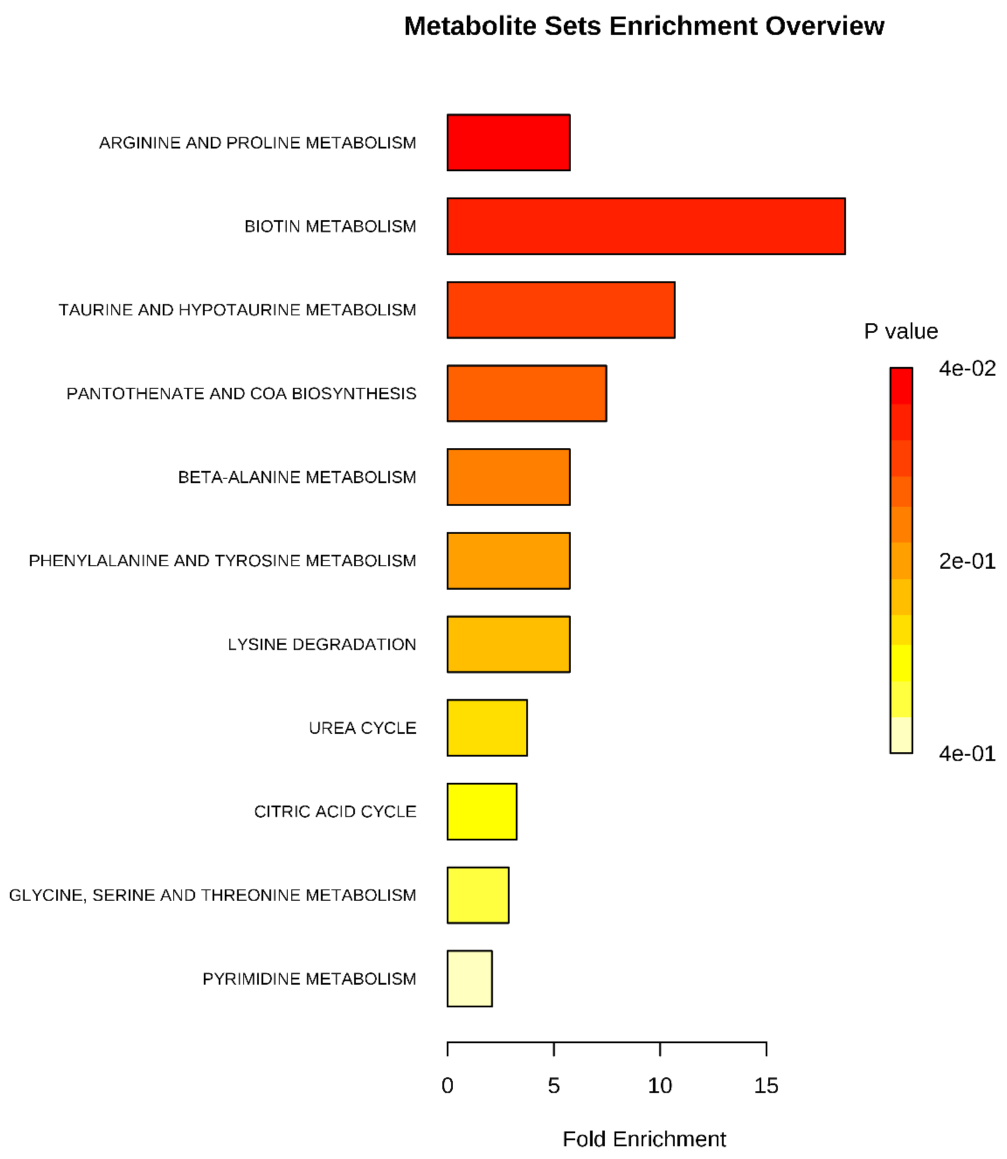

Figure S4. Pathway enrichment analysis of metabolites that were significantly linear (including combined rhythmic and linear metabolites) in group-level analyses. Unadjusted p-values are represented. Generated by MetaboAnalyst 3.0.

Table S8. Results of the pathway enrichment analysis of identified individual-level rhythmic (including combined rhythmic and linear) metabolites from the targeted matrix. The table shows the pathway involved, the total number of metabolites in that pathway, the number of significantly rhythmic metabolites in that pathway and the raw and false discovery rate (FDR) adjusted p values. Results were generated from MetaboAnalyst 3.0.

**Pathway**	**Total metabs. in pathway**	**# sig. metabs. in pathway**	**Raw p**	**FDR adjusted p**
Citric acid cycle	23	9	0.000107	0.00847
Urea cycle	20	8	0.000226	0.00892
Methionine metabolism	24	6	0.0201	0.371
Galactose metabolism	25	6	0.0244	0.371
Phenylalanine and tyrosine metabolism	13	4	0.0275	0.371
Gluconeogenesis	27	6	0.035	0.371
Starch and sucrose metabolism	14	4	0.0358	0.371
Nucleotide sugars metabolism	9	3	0.0453	0.371
RNA transcription	9	3	0.0453	0.371
Biotin metabolism	4	2	0.0469	0.371
Purine metabolism	45	8	0.0536	0.385
Ammonia recycling	18	4	0.0819	0.477
Glutamate metabolism	18	4	0.0819	0.477
Arginine and proline metabolism	26	5	0.0905	0.477
Aspartate metabolism	12	3	0.0964	0.477
Insulin signalling	19	4	0.0965	0.477
Intracellular signalling through prostacyclin receptor and prostacyclin	6	2	0.104	0.481
Beta-alanine metabolism	13	3	0.117	0.487
Lysine degradation	13	3	0.117	0.487
Glycolysis	21	4	0.129	0.5
Taurine and hypotaurine metabolism	7	2	0.136	0.5
Beta oxidation of very long chain fatty acids	14	3	0.139	0.5
Mitochondrial electron transport chain	15	3	0.163	0.559
Malate-aspartate shuttle	8	2	0.171	0.562
Propanoate metabolism	18	3	0.239	0.703
Pentose phosphate pathway	18	3	0.239	0.703
Betaine metabolism	10	2	0.243	0.703
Valine, leucine and isoleucine degradation	36	5	0.249	0.703

Inositol metabolism	19	3	0.266	0.724
Pyruvate metabolism	20	3	0.293	0.771
Glucose-alanine cycle	12	2	0.317	0.808
Intracellular signalling through FSH receptor and follicle stimulating hormone | intracellular signalling through LHCGR receptor and luteinizing hormone/choriogonadotropin	4	1	0.329	0.812
Catecholamine biosynthesis	5	1	0.393	0.817
Excitatory neural signalling through 5-HTR 4 and serotonin | excitatory neural signalling through 5-HTR 7 and serotonin | excitatory neural signalling through 5-HTR 6 and serotonin	5	1	0.393	0.817
Corticotropin activation of cortisol production	5	1	0.393	0.817
Vasopressin regulation of water homeostasis	5	1	0.393	0.817
Intracellular signalling through histamine H2 receptor and histamine	5	1	0.393	0.817
Intracellular signalling through PGD2 receptor and prostaglandin D2	5	1	0.393	0.817
Pyrimidine metabolism	36	4	0.45	0.89
Alanine metabolism	6	1	0.451	0.89
Intracellular signalling through adenosine receptor A2A and adenosine | intracellular signalling through adenosine receptor A2B and adenosine	7	1	0.503	0.97
Butyrate metabolism	9	1	0.594	1
Pantothenate and CoA biosynthesis	10	1	0.633	1
Ketone body metabolism	10	1	0.633	1
Tyrosine metabolism	38	3	0.718	1
Glycine, serine and threonine metabolism	26	2	0.725	1
Oxidation of branched chain fatty acids	14	1	0.755	1
Sphingolipid metabolism	15	1	0.778	1
Amino sugar metabolism	15	1	0.778	1
Fructose and mannose degradation	18	1	0.837	1
Tryptophan metabolism	34	2	0.851	1
Fatty acid metabolism	29	1	0.947	1
Steroid biosynthesis	31	1	0.957	1
Bile acid biosynthesis	49	1	0.993	1

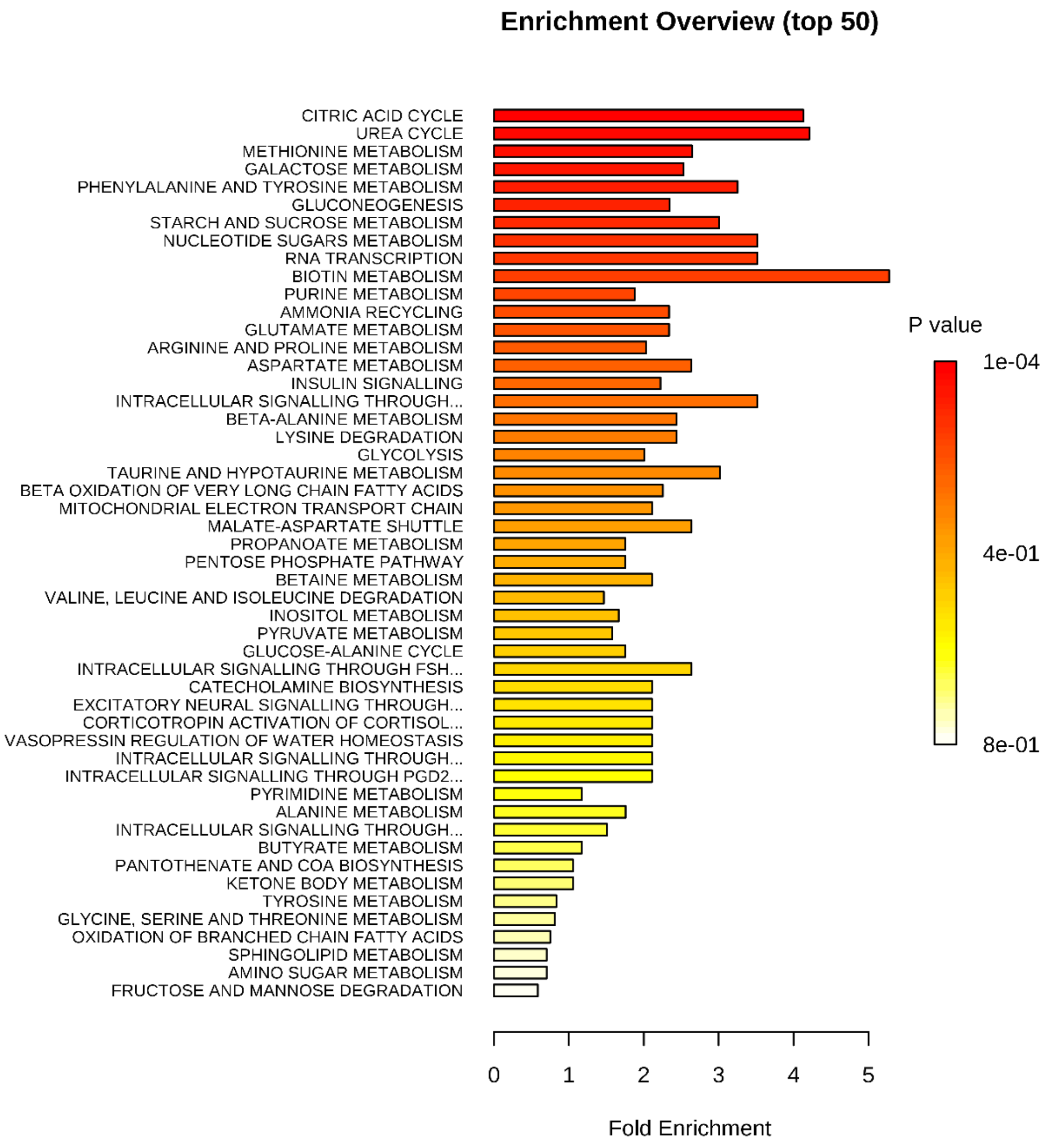

Figure S5. Pathway enrichment analysis of metabolites that were significantly rhythmic (including combined rhythmic and linear metabolites) in individual-level analyses. Unadjusted p-values are represented. Generated by MetaboAnalyst 3.0.

Table S9. Results of the pathway enrichment analysis of identified individual-level linear (including combined rhythmic and linear) metabolites from the targeted matrix. The table shows the pathway involved, the total number of metabolites in that pathway, the number of significantly linear metabolites in that pathway and the raw and false discovery rate (FDR) adjusted p values. Results were generated from MetaboAnalyst 3.0.

**Pathway**	**Total metabs. in pathway**	**# sig. metabs. in pathway**	**Raw p**	**FDR adjusted p**
Citric acid cycle	23	10	2.41E-06	0.00019
Urea cycle	20	8	5.83E-05	0.0023
Malate-aspartate shuttle	8	4	0.00195	0.0337
Beta-alanine metabolism	13	5	0.00207	0.0337
Galactose metabolism	25	7	0.00213	0.0337
Alanine metabolism	6	3	0.00793	0.104
Ammonia recycling	18	5	0.0101	0.107
Glucose-alanine cycle	12	4	0.0108	0.107
Arginine and proline metabolism	26	6	0.0126	0.11
Gluconeogenesis	27	6	0.0152	0.12
Starch and sucrose metabolism	14	4	0.0194	0.139
Nucleotide sugars metabolism	9	3	0.028	0.184
Propanoate metabolism	18	4	0.0467	0.263
Glutamate metabolism	18	4	0.0467	0.263
Insulin signalling	19	4	0.0557	0.293
Aspartate metabolism	12	3	0.0618	0.305
Pyruvate metabolism	20	4	0.0656	0.305
Phenylalanine and tyrosine metabolism	13	3	0.0759	0.318
Glycolysis	21	4	0.0764	0.318
Beta oxidation of very long chain fatty acids	14	3	0.0914	0.361
Mitochondrial electron transport chain	15	3	0.108	0.406
Methionine metabolism	24	4	0.114	0.408
Cysteine metabolism	8	2	0.126	0.434
Pentose phosphate pathway	18	3	0.164	0.539
Betaine metabolism	10	2	0.183	0.559
Inositol metabolism	19	3	0.184	0.559
Biotin metabolism	4	1	0.281	0.822
Valine, leucine and isoleucine degradation	36	4	0.315	0.888
Catecholamine biosynthesis	5	1	0.338	0.89

Glycine, serine and threonine metabolism	26	3	0.338	0.89
Intracellular signalling through prostacyclin receptor and prostacyclin	6	1	0.391	0.995
Taurine and hypotaurine metabolism	7	1	0.439	1
Purine metabolism	45	4	0.483	1
Butyrate metabolism	9	1	0.525	1
Pyrimidine metabolism	36	3	0.553	1
Glutathione metabolism	10	1	0.563	1
Pantothenate and CoA biosynthesis	10	1	0.563	1
Ketone body metabolism	10	1	0.563	1
Tyrosine metabolism	38	3	0.591	1
Histidine metabolism	11	1	0.598	1
Lysine degradation	13	1	0.66	1
Glycerolipid metabolism	13	1	0.66	1
Oxidation of branched chain fatty acids	14	1	0.687	1
Amino sugar metabolism	15	1	0.712	1
Fructose and mannose degradation	18	1	0.776	1
Fatty acid metabolism	29	1	0.912	1
Steroid biosynthesis	31	1	0.926	1
Tryptophan metabolism	34	1	0.943	1

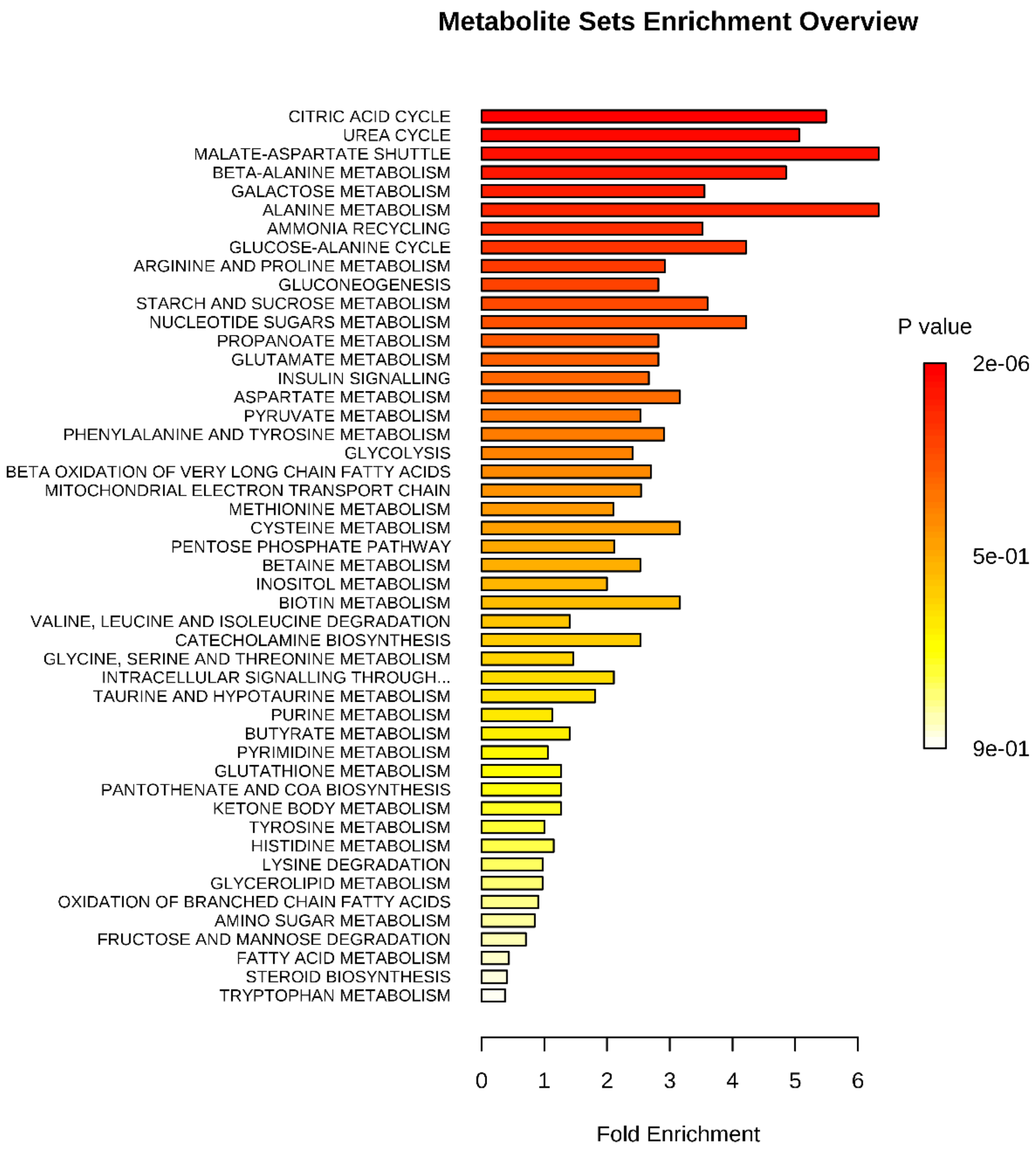

Figure S6. Pathway enrichment analysis of metabolites that were significantly linear (including combined rhythmic and linear metabolites) in individual-level analyses. Unadjusted p-values are represented. Generated by MetaboAnalyst 3.0.
